# Nutrient Status and perceptions of energy and macronutrient intake in a Group of Collegiate Female Lacrosse Athletes

**DOI:** 10.1186/s12970-019-0314-7

**Published:** 2019-10-15

**Authors:** Andrew R. Jagim, Hannah Zabriskie, Brad Currier, Patrick S. Harty, Richard Stecker, Chad M. Kerksick

**Affiliations:** 1 Sports Medicine, Mayo Clinic Health System, 109 Theater Road, Onalaska, WI 54650 USA; 2 Exercise and Performance Nutrition Laboratory, Lindenwood University, St. Charles, MO USA

**Keywords:** Nutrition, Calories, Energy, Macronutrients, Females, Athletes, Energy balance, Energy expenditure, Energy availability

## Abstract

**Background:**

The purpose of this study was to compare nutritional intakes against recommended values as well as between the perceived intake and needs of female lacrosse players.

**Methods:**

Twenty female NCAA Division II lacrosse players (20.0 ± 1.7 yrs., 169.7 ± 6.4 cm; 69.9 ± 10.7 kg; 27.5 ± 3.3% fat) completed a four-day monitoring period during in-season. Athletes were outfitted with an activity monitor over four consecutive days and completed four-day food records to assess total daily energy expenditure (TDEE) and dietary intake. Body composition was assessed and used to calculate recommended dietary intakes. Actual intake was self-reported using a commercially available food tracking program (MyFitnessPal©, USA). Daily average values were calculated for total and relative energy, protein, carbohydrate, and fat intake. These values were then compared to published nutritional recommendations established by the International Society of Sports Nutrition. Appropriate pairwise comparisons were made depending on the normality of the distribution.

**Results:**

Athletes ate significantly less than recommended values for energy, carbohydrates and protein. (*p* < 0.001). Significant discrepancies (*p* < 0.001) were also observed between perceptions of intake versus actual intake.

**Conclusions:**

Athletes significantly underestimated perceived intake of dietary fat and carbohydrate when compared to perceived needs. Massive standard deviations and ranges were observed, suggesting that some athletes lack a basic understanding of their daily needs. Results from this data suggest that collegiate athletes lack appropriate understanding of basic nutrition needs and could benefit from basic nutrition education as it pertains to their health and performance.

## Background

Dietary practices have a profound impact on athlete health and performance [1, 2]. Due to increased physical demands, athletes achieve daily energy expenditures that require above-average energy and macronutrient intakes to sustain training, enhance recovery, and maintain performance [1]. To address these specialized dietary requirements, professional organizations such as the International Society of Sports Nutrition (ISSN) and the International Olympic Committee (IOC) have established dietary recommendations for athletes [1–3]. However, many athletes struggle to follow dietary recommendations, and several studies have shown that actual nutrient intakes of collegiate athletes and reported energy and macronutrient intake levels fall below the recommended daily allowance (RDA) or recommendations made by professional organizations [4–8]. These findings should be interpreted in light of the fact that athletes may have higher energy, macronutrient, and micronutrient requirements than RDA-derived values, and therefore, their nutrient intakes may need to be even higher than RDA values [1]. Previous research has indicated that female athletes are at particular risk for nutritional deficiencies resulting from a high volume of training coupled with inadequate energy and macronutrient intake [9, 10]. Such deficiencies can compromise performance, impair recovery, negatively influence endocrine function, and increase susceptibility to injuries and illness [10].

Factors such as inadequate nutrition knowledge, logistical challenges (i.e. traveling, time spent practicing, accessibility etc.), physique requirements, social pressures, and inadequate financial resources are often listed as key barriers to acceptable nutrition within elite athletes [11, 12]. Furthermore, previous [5, 12–16] reports indicate that athletes struggle to correctly identify recommended levels of macronutrient intakes for their sport or correctly answer questions about basic nutrition knowledge, supplements, weight management, and hydration [13, 16]. A failure to understand how dietary requirements fluctuate with the changing demands of training and competition may result in situations where athletes are chronically under-fueled. Unfortunately, even well-informed athletes may not translate nutrition knowledge to a sufficient dietary intake due to the barriers outlined previously. Moreover, an athlete’s perceptions of their diet may not align with their nutritional requirements or their actual intake. These issues are likely further magnified at the collegiate level where nutrition-based support services or educational resources are often not available to student athletes.

Currently, there is limited research evaluating how effectively female team sport athletes meet sport-specific nutritional recommendations. Furthermore, there is a paucity of research exploring the accuracy of athletes’ perception of their energy and macronutrient needs, as well as their perceived dietary intake compared to their actual consumption. Therefore, the purpose of this study was two-fold: 1) To compare calculated ISSN recommendations for daily energy and macronutrient intake to actual in-season dietary intake of female lacrosse players; 2) To identify the discrepancies that exist in female collegiate athletes between their perceived energy and macronutrient needs as well as between their perceived and actual intake of energy and macronutrients.

## Methods

### Experimental design

All athletes were outfitted with an activity monitor (Acti-Heart, CamNTech, Inc.) during in-season competition and team activities over four consecutive days (worn continuously). The monitoring period consisting of 2 weekdays and 2 weekend days during May of the 2018 season and was used to assess total daily energy expenditure (TDEE). Participants were assessed for body composition, which was used to calculate recommended intake values [1, 2]. Participants also recorded dietary intake (food and fluid) during the four-day monitoring period using a commercially available food tracking program. Daily average values were calculated for total and relative energy, protein, carbohydrate, and fat intake. These values were then compared to nutritional recommendations provided in position stands and summary statements put forth by the ISSN [2] and American College of Sports Medicine (ACSM) [1]. To avoid bias, we identified energy intake recommendations for low, middle, and high levels of activity (40, 50, and 60 kcals/kg/day, respectively). The same methods were used to determine intakes for carbohydrate (4, 6, and 8 g/kg/day), protein (1.4, 1.6, and 1.8 g/kg/day), and fat (15, 25, and 35% calories), respectively. Recommendations for energy intake were compared to measured TDEE from activity monitors. In addition, each athlete recorded their perceived nutrient needs and their perceived nutrient intake over the collection period by completing a questionnaire.

### Study participants

Twenty female NCAA Division II Lacrosse players (20.0 ± 1.7 years, 169.7 ± 6.4 cm; 69.9 ± 10.7 kg; 27.5 ± 3.3% body fat) completed all aspects of testing. All athletes were medically cleared and participated in all team activities during the study period. Team members who were not able to participate in all team activities were excluded from the study. Prior to testing, all athletes provided written consent and the study protocol was approved by the Lindenwood University Institutional Review Board.

### Dietary intake

Dietary energy intake was assessed from four-day diet logs completed during the same days that energy expenditure was monitored. Subjects were given food log packets that illustrated how to accurately record portion sizes of various foods and beverages consumed. Athletes logged all calorie-containing food and beverages for four consecutive days using the MyFitnessPal smartphone application (MyFitnessPal©, USA). Four-day averages were computed for energy, carbohydrate, protein, and fat intake and both raw values and values normalized to body mass in kilograms were used in the analysis.

### Resting energy expenditure

All resting energy expenditure (REE) measures were completed using a ParvoMedics TrueOne 2400 metabolic measurement system (Sandy, UT). Each morning the indirect calorimetry system was calibrated to ensure that variations in measured oxygen and carbon dioxide and the flow rate were less than 2% different than the previous calibration. A clear plastic hood and drape was placed over each participant’s head and shoulders with the flow rate on the dilution pump set to maintain approximately 0.8–1.2% carbon dioxide in the expired gases. Study participants remained awake and motionless in a supine position for 20–25 min while data were collected at 1-min intervals. The recorded data were visually inspected to identify a 5-min window during the final 10 minutes of data collection where VO_2_ (in L/min) changed less than 5% to identify a daily average of REE (in kcal/day). Participants were instructed to fast from all energy-containing foods and fluids for a minimum of 8 hours prior to the test and did not exercise or perform physical activity for 24 h prior to the test. All REE assessments occurred within 2 weeks of activity energy expenditure assessment and self-reporting of dietary intake.

### Body composition (DEXA)

Body composition assessments were completed using dual-energy x-ray absorptiometry (DEXA). To standardize testing conditions, participants observed an 8-h fast of energy-containing food and fluid and avoided exercise for at least 24 h [17] prior to assessments. All DEXA scans occurred within 2 weeks before or after activity energy expenditure assessment and self-reporting of dietary intake. Calibration procedures were completed each day before testing, and all DEXA scans were completed using the Discovery DEXA System (HOLOGIC, Inc., Bedford, MA) and analyzed using its accompanying software (Hologic APEX Software, Version 4.5.3, HOLOGIC, Bedford, MA) to determine whole-body levels of bone, fat, and fat-free masses along with body fat percentages. Previous test-retest reliability analysis of these procedures yielded intra-class correlation coefficients of > 0.998.

### Activity energy expenditure

In-season activity energy expenditure was assessed during the same four-day period used for dietary intake assessment, consisting of two weekdays and two weekend days. Whenever possible, the assessment period contained a game day, an off day, and two practice days using only accelerometer data from the physical activity monitors (Acti-Heart, CamNTech, Inc., Boerne, TX). Each monitor was worn on the left side of the athlete’s chest below their left breast. The monitors were attached at the level of the xiphoid at the anterior midline and laterally positioned at the anterior axillary line using standard ECG electrodes. Both electrode locations were adjusted to ensure the lead wire was parallel to the ground. The Acti-Heart software computes activity energy expenditure, predicts resting metabolic rate using the equation of Schofield [18], and assigns a value for thermic effect of food that is fixed at 10 % of the computed TDEE. TDEE and activity energy expenditure (AEE) values were used to create an average of TDEE and AEE over the entire 4-day period. Previously published work by Assah et al. [19, 20] in a large group of free-living adults revealed that the monitors were valid in comparison to doubly labeled water.

### Perceptions of dietary intake and dietary needs

A brief questionnaire was developed to compare the athletes’ perceptions of their nutrient needs to ISSN derived energy and macronutrient intake recommendations based on the requirements of their sport. A secondary aim of the questionnaire was to assess each athlete’s perceived energy and macronutrient intake on a typical day and then compared to the established dietary guidelines for their sport.

### Statistical analysis

From the original 22 participants recruited to complete this study, two participants were removed from the analysis due to noncompliance with the testing protocol. Three additional participants did not complete perceived nutrition intake questionnaires. Thus, a sample size of 17 was used for all calculations involving perceived intake, while a sample size of 20 was used for all other calculations. To aid in understanding of the magnitude of discrepancy with perceived needs and intake, only raw values were used for computations (i.e., kcals/day and grams/day). A *p*-value of <0.05 was used to determine statistical significance. SPSS V.25 for Windows (Armonk, NY) and Microsoft Excel (Seattle, WA) were used to complete all statistical analyses. All normally distributed data are presented as means ± standard deviations and all non-normally distributed data are presented as median ± interquartile range (IQR). The Shapiro-Wilk test was used to determine normality. When normality was confirmed, paired samples t-tests were used to assess differences between groups. When the normality assumption was violated, Wilcoxon Signed Rank tests were used to assess differences between the non-normally distributed variables.

## Results

Significant differences (*p* < 0.001) were observed between all energy and macronutrient recommendations when compared to actual intakes. These differences were present for both total and relative daily values. For energy and all macronutrient recommendations, athletes consumed well below the recommendations (Table 1). Absolute TDEE in this study was determined to be 2582 ± 303 kcals/day, (95% CI: 2441, 2724) while relative energy expenditure was 37.9 ± 4.7 kcal/kg/day (95% CI: 35.7, 40.1). The ISSN has provided general energy intake recommendations of 40, 50, and 60 kcals/kg/day for athletes participating in low, medium, and high levels of training volume, respectively. Using the measured body mass levels of athletes in our cohort, these recommended energy intake values translated into 2756 ± 403 kcals/day (95% CI: 2567, 2945), 3445 ± 504 kcals/day (95% CI: 3209, 3681), and 4134 ± 605 kcals/day (95% CI: 3851, 4417), respectively for the low, medium, and high energy intake recommendations. When compared to self-reported dietary intake (2161 ± 392 kcals/day), the athletes in the current study reported the intake of less energy than all recommended levels. When compared to the lowest recommended intake, the mean difference between this recommended intake amount and the self-reported intake amount was − 595 ± 605 kcals/day (95% CI: − 878, − 312 kcals/day). The mean differences between the medium and high recommended energy intake levels and the self-reported energy intake level were − 1284 ± 685 kcals/day (− 1604, − 963 kcals/day) and − 1973 ± 771 kcals/day (− 2333, − 1612 kcals/day), respectively. All recommended energy intake levels were significantly different than the reported energy intake levels (*p* < 0.001).

**Table 1:** Comparison of recommended dietary intake versus actual intake (*n* = 20)

	Actual Intake*	Recommended§		Delta Intake (Actual – Recommended)	*p* value
Total Energy Intake (kcal/d)	2161 ± 392 (1978, 2344)	Low	2756 ± 403 (2567, 2945)	− 595 ± 605 (− 878, − 312)	<0.001
		Moderate	3445 ± 504 (3209, 3681)	− 1284 ± 685 (− 1604, − 963)	<0.001
		High	4134 ± 605 (3851, 4417)	−1973 ± 771 (− 2333, − 1612)	<0.001
Relative Energy Intake (kcal/kg/d)	32.1 ± 7.9 (28.4, 35.6)	Low	40		
		Moderate	50		
		High	60		
Total CHO Intake (g/d)	236 ± 74 (201, 270)	Low	275.6 ± 40.3 (257, 294)	− 40.0 ± 83.4 (− 79, − 0.94)	0.05
		Moderate	413.4 ± 60.5 (385, 442)	− 178 ± 94 (− 222, − 134)	<0.001
		High	551.2 ± 80.7 (514, 589)	− 316 ± 108 (− 366, − 265)	<0.001
Relative CHO Intake (g/kg/d)	3.48 ± 1.19 (2.92, 4.03)	Low	4.0		
		Moderate	6.0		
		High	8.0		
Total PRO Intake (g/d)	78.8 ± 19.6 (69.6, 88.0)	Low	96.5 ± 14.1 (90, 103)	− 17.7 ± 28.2 (− 31.9, − 4.5)	0.011
		Moderate	110.2 ± 16.1 (103, 118)	−31.4 ± 29.8 (− 45.4, − 17.5)	<0.001
		High	124.0 ± 18.1 (116, 133)	−45.2 ± 31.4 (−59.9, − 30.5)	<0.001
Relative PRO Intake (g/kg/d)	1.18 ± 0.38 (1.00, 1.36)	Low	1.4		
		Moderate	1.6		
		High	1.8		
Total Fat Intake (g/d)	87.9 ± 22.8 (77.3, 98.6)	Low	36.0 ± 6.5 (33.0, 39.1)	51.9 ± 19.7 (42.7, 61.1)	<0.001
		Moderate	60.0 ± 10.9 (54.9, 65.1)	27.9 ± 18.7 (19.1, 36.6)	<0.001
		High	84.1 ± 15.2 (76.9, 91.2)	3.9 ± 18.6 (−4.9, 12.6)	0.37
Relative Fat Intake (g/kg/d)	1.31 ± 0.41 (1.11, 1.50)	Low	15%		
		Moderate	25%		
		High	35%		

§Recommend values are derived through a combination of published review articles [1, 2] and clinical experience. Delta Intake = Actual intake – Recommended intake. All variables exhibited normal distributions using Shapiro-Wilk tests (*p* > 0.05). Data presented as mean ± SD with the 95% confidence interval presented in parentheses below the mean ± SD. * = As outlined in statistical analysis section, dietary intake values reported in Table 1 and Table 2 are expected to be slightly different due to the removal of three participants who failed to complete all of the required perceived nutrition assessment

In addition, discrepancies were found between the actual reported carbohydrate intake and recommended intake. Paired samples t-tests between the reported carbohydrate intake (236 ± 73 g/day, 95% CI: 201, 270 g/day) and the low, moderate and high recommended amounts were all statistically significant (Table 1). Similarly, paired samples t-tests between the reported protein intake (79 ± 20 g/day, 95% CI: 70, 88 g/day) and the low, moderate and high recommended amounts were all statistically significant (Table 1). Finally, paired samples t-tests between the reported fat intake (88 ± 23 g/day, 95% CI: 77, 99 g/day) and the low and moderate recommended amounts were found to be statistically significant, while no significant difference was found between actual fat intake and the high recommended amount (*p* = 0.37).

Because the perceived needs and intake data were not normally distributed, we used Wilcoxon Signed Rank tests were used to assess differences between perceived needs and perceived intake as well as between perceived intake and actual intake. As seen in Table 2, widespread variability existed within the perceived needs and intake data resulting in large deviations from normality. Significant differences were observed between the perceived needs and perceived intakes for absolute carbohydrate (*p* = 0.004) and absolute fat (*p* = 0.007). No significant differences were observed between perceived intake and actual intake for energy, carbohydrate, protein, or fat intake. All perceived needs, perceived intake and actual dietary intake data are provided in Table 2.

**Table 2:** A comparison of perceived versus actual dietary intake (*n* = 17)

	Perceived Needs	Perceived Intake	Delta Perceived *p-value*	Actual Intake	Delta Intake *p-value*
Energy Intake (kcals/day)	2000 ± 300	2214 ± 679 ^a^ (1,866, 2564)	−30.8 ± 563^a^‡ (− 320, 259) *0.42*	2137 ± 418 ^a^ (1,922, 2351)	78.0 ± 693 ^a^‡ (− 278, 434) *0.65*
Carbohydrate Intake (grams/day)	80 ± 213	150 ± 280	− 129 ± 228* *0.004*	242 ± 73 ^a^ (204, 279)	−30 ± 240 ‡ *0.33*
Protein Intake (grams/day)	45 ± 41	30 ± 32.5	−33.8 ± 42.3* *0.39*	77.7 ± 20.4 ^a^ (67, 88)	−42.8 ± 42.3 ‡ *0.98*
Fat Intake (grams/day)	30 ± 40	50 ± 63	22.9 ± 120* (− 84.6, 38.8) *0.007*	86.1 ± 22.4 ^a^ (75, 98)	62.1 ± 306 ‡ (− 95, 219) 0.42

^a^= Data is normally distributed and presented as means ± standard deviation with the 95% confidence interval in parentheses. Delta perceived = perceived needs – perceived intake. Delta intake = perceived intake – actual intake. * = *p*-value from Wilcoxon Signed Rank Test (non-normally distributed variables). ‡ = p-value from paired samples t-test (normally distributed data). When one value was non-normally distributed and another variable was normally distributed, a paired samples t-test was used to assess differences between the two means

## Discussion

The primary aim of the current study was to evaluate the ability of collegiate female lacrosse players to meet nutritional recommendations established by the ISSN. Secondary aims were to investigate the accuracy of the athletes’ perception of their nutritional requirements and to compare their perceived intake to their actual dietary intake. This investigation found that the athletes failed to meet energy and macronutrient recommendations put forth by professional organizations. However, significant deviations were observed when estimated energy requirements were based off the “moderate” or “high” activity levels and compared to the measured TDEE. In this respect, the predicted daily energy requirement that corresponded with the low activity level (40 kcal/kg of bodyweight) was similar to the mean (SD) measured TDEE of 37.9 ± 4.7 kcals/kg of bodyweight. This level of total daily energy expenditure appears to remain fairly consistent, even throughout the duration of a season in female lacrosse players [21] with average game energy expenditures ranging between 800 and 1000 kcals/game. Even when using a conservative “low” activity level to estimate energy requirements, the athletes in the current study still failed to meet energy requirements, as their mean energy intake was 32.1 ± 7.9 kcal/kg. Previous work has also reported an inability of athletes to meet nutritional guidelines for their sport [4, 8, 22–25], as consuming adequate energy and carbohydrates appears to be a challenge for many athletes [26]. Beals and colleagues [24] noted that female adolescent volleyball players largely failed to meet recommended intakes for energy, carbohydrates and protein and presented with several micronutrient deficiencies. Similarly, Clark et al. [7] determined that many female collegiate soccer players also failed to meet carbohydrate recommendations, but did meet the Dietary Reference Intake for energy intake for individuals with an “active lifestyle” (37 kcal/kg/day). This is in alignment with the results of the current study, as this recommended value is near the ISSN recommendation of 40 kcal/kg/day that best aligned with the TDEE exhibited by the current players during training (37.9 ± 4.7 kcal/kg/day). Valliant et al. [23] also reported that a group of female collegiate volleyball players failed to meet dietary requirements for energy, carbohydrate, and protein.

It is not well understood whether such nutritional issues are a result of a lack of knowledge, a misunderstanding of dietary behaviors, or the result of other negative influences and bad habits. In the current study, widespread discrepancies were found between athletes’ perceptions of their energy and macronutrient needs and their actual dietary intakes as well as between their perceived nutrient intakes and actual intakes. In particular, the athletes’ perceived daily carbohydrate and fat needs and perceived dietary intakes displayed massive standard deviations and robust ranges, suggesting that some athletes lack even the most basic understanding of their daily nutritional needs. Moreover, the athletes in the current study perceived their protein needs to be less than what they were consuming. Disparities were also present between an athlete’s perceptions of their macronutrient needs when compared to recommended intakes as well as between their perceived nutrient intake and their actual intake. However, the athletes did seem to possess a better grasp of their energy needs, as no differences were found between their perceived energy intake, perceived energy need, and actual energy intake.

Results from this study suggest that collegiate athletes lack appropriate understanding of basic nutrition needs and could likely benefit from basic nutrition education. Targeted sport nutrition education interventions have been shown to exert a positive impact on the nutritional knowledge and dietary intake of collegiate athletes [23, 27, 28]. For example, Abood et al. [28] observed that an 8-week (1 h. per week) nutrition education program improved nutrition knowledge and resulted in subsequent positive dietary changes in NCAA Division I female soccer players, though deficiencies in total energy and macronutrient intake were still observed. Similarly, Valliant et al. [23] reported a significant improvement in sports nutrition knowledge as well as total energy and macronutrient intake in NCAA Division I female Volleyball players following an off-season nutrition intervention program. Most recently, Rossi et al. [27] observed significant improvements in nutritional intake, body composition, and performance following a single 90-min sport nutrition education intervention in NCAA Division I baseball players. However, an athlete’s nutrition knowledge may only slightly influence dietary intake, as logistical reasons such as time, cooking skills, and financial constraints could prevent a well-informed athlete from eating effectively [11, 12].

It is tempting to simply recommend that all universities employ a full-time registered dietician or sports nutritionist, as access to these professionals can improve nutrition knowledge [29, 30]. For many athletic departments, however, these positions are not fiscally possible. Consequently, athletes at these schools tend to rely upon their sport coaches and/or strength and conditioning professionals who may not have enough knowledge to provide appropriate guidance [16]. Even with the available nutritional support staff or educational resources, adherence to nutritional guidelines may still be an issue, as many collegiate athletes are in a unique transitional stage in their lives where they may be tasked with purchasing, preparing, and making decisions about food for the first time. Thus, it is worth noting that even if the athletes have an appropriate level of understanding of nutrition, they may still opt for lower quality options or fail to realize they are not meeting their perceived dietary needs as previously observed and supported by the results of the current study [12].

The use of self-reported dietary intakes is a limitation of the current study as previous research has indicated that dietary logs may be susceptible to underreporting in athlete populations [31]. Additionally, little information is currently available regarding the physiological demands of women’s lacrosse. As a result, there are not sport-specific nutritional recommendations available for women’s lacrosse which is also a limitation of the current study. Comparisons are often made between soccer and lacrosse as they are both intermittent team-based field sports consisting of short bursts of high-intensity sprinting intermixed with jogging [32]. Female lacrosse players also exhibit anthropometric and fitness characteristics that are similar to women’s basketball [32].Therefore the nutritional recommendations used in the current study should reflect the nutritional requirements of collegiate female lacrosse players as the ISSN recommendations are designed to meet the needs of an athlete training at this level [2, 33]. Furthermore, we provided ranges of nutritional recommendations to avoid any bias and present multiple tiers of nutritional requirements based on changes in training level. Future research should focus on examining the physiological demands and sport-specific nutritional requirements of team sports, especially in female athletes.

## Conclusions

The results of this study clearly indicate that many collegiate athletes may lack appropriate understanding of basic nutrition needs and could benefit from basic nutrition education, which would likely reduce training related injuries and optimize performance. Because proper sport nutrition is a vital to ensure peak performance and recovery, qualified professionals face the challenge to educate athletes regarding the benefits of a nutrient-dense diet that meets appropriate caloric needs. As smaller institutions frequently do not have the resources to hire full-time nutritional staff, it may be beneficial for coaches to offer a nutrition-education program to ensure their players are meeting the energy requirements for their body size and level of training.

## Data Availability

The datasets used and/or analyzed during the current study are available from the corresponding author on reasonable request.

## Abbreviations

AEE: activity energy expenditure
DEXA: dual-energy x-ray absorptiometry
ISSN: International Society of Sports Nutrition
NCAA: National Collegiate Athletic Association
RDA: recommended daily allowance
REE: resting energy expenditure
TDEE: total daily energy expenditure
